# Single-shot quantitative phase microscopy with color-multiplexed differential phase contrast (cDPC)

**DOI:** 10.1371/journal.pone.0171228

**Published:** 2017-02-02

**Authors:** Zachary F. Phillips, Michael Chen, Laura Waller

**Affiliations:** 1 Graduate Group in Applied Science and Technology, University of California, Berkeley, United States of America; 2 Dept. of Electrical Engineering and Computer Sciences, University of California, Berkeley, United States of America; Tufts University, UNITED STATES

## Abstract

We present a new technique for quantitative phase and amplitude microscopy from a single color image with coded illumination. Our system consists of a commercial brightfield microscope with one hardware modification—an inexpensive 3D printed condenser insert. The method, color-multiplexed Differential Phase Contrast (cDPC), is a single-shot variant of Differential Phase Contrast (DPC), which recovers the phase of a sample from images with asymmetric illumination. We employ partially coherent illumination to achieve resolution corresponding to 2× the objective NA. Quantitative phase can then be used to synthesize DIC and phase contrast images or extract shape and density. We demonstrate amplitude and phase recovery at camera-limited frame rates (50 fps) for various *in vitro* cell samples and c. elegans in a micro-fluidic channel.

## Introduction

Quantitative Phase Imaging (QPI) involves recovering the complex field of a sample—both amplitude and phase. This enables label-free and stain-free optical imaging of biological samples *in vitro*. In contrast to *qualitative* phase imaging methods, such as Zernike phase contrast (PhC) [1]) and Differential Interference Contrast (DIC), *quantitative* methods recover the phase delay caused by the sample, decoupled from absorption information. Modifications of PhC [2] and DIC [3] can make these setups quantitative, at a cost of requiring multiple images. More commonly, QPI methods use interferometry with coherent illumination and a reference beam [4–6], making them expensive and sensitive to misalignment and vibrations.

Amongst the wide array of existing QPI methods, several are single-shot techniques. Off-axis holography interferes the sample beam with a tilted reference beam, then recovers phase by Fourier filtering [7]. Parallel phase-shifting can spatially multiplex several holograms within a single exposure via an array of polarizers [8]. Single-shot QPI add-ons based on amplitude gratings work with commercial microscopes, replacing the traditional camera module [9, 10]. Another add-on option uses two cameras to capture defocused images which can then be used to solve the Transport of Intensity Equation (TIE) [11]. Alternatively, if chromatic aberrations are large enough, they can enable single-shot color TIE [12] without any hardware changes. This concept of color multiplexing is similar to that used in photographic depth ranging [13]. All of these methods require some level of spatial or temporal coherence, limiting resolution. We seek here a single-shot QPI method that achieves the spatially incoherent resolution limit.

Differential Phase Contrast (DPC) [14–17] is a partially coherent QPI technique that requires multiple images. Each is captured using a different asymmetric half-circle source pattern, which shifts the sample’s spectrum in Fourier space. Thus, a half circle source and its complement will cause the pupil function to crop opposite sides of the sample’s spectrum. Since imaginary information is encoded in Fourier asymmetry, these images can be used to recover phase. Assuming a linearized model for a weakly scattering sample, the inverse problem becomes a single-step deconvolution process [15, 17]. DPC recovers both amplitude and phase with resolution up to the incoherent resolution limit (2× better than coherent methods). Practically, the illumination switching can be done quickly and at low cost with an LED array [16–18]. At least two complementary source patterns are required, but generally 4 patterns (top, bottom, left, right half-circles) are used to avoid missing frequencies. The DPC method was recently extended to color multiplexing [19], where the 4 source patterns were encoded into two images by using a color camera in combination with a color LED array. Similarly, color photometric stereo has been used for retrographic surface profiling of large objects using off-axis color illumination in reflection mode [20].

Our method, termed color Differential Phase Contrast (cDPC), requires only a *single* color image for multiplexing source patterns. The three RGB source color channels are used to display three different half-circle source patterns. A 4th image is not needed, since it can be synthesized by taking the sum of two images acquired with opposite half-circle illuminations (a synthetic brightfield image) and subtracting that of a 90 degree rotated half-circle source. Thus we require only 3 illumination patterns and 3 measurements, which are collected in a single shot using a RGB Bayer filter sensor. We start by implementing the source pattern in an LED array microscope, which offers many imaging modalities in one platform [16–18, 21–24]. However, our configuration does not require a dynamic source. We instead design a static multi-color filter to be placed in the condenser back focal plane, assuming Köhler illumination. Both configurations simplify hardware and reduce costs significantly as compared to phase contrast or DIC, while providing quantitative phase, which is more general and can be used to synthesize both of the aforementioned methods digitally [25].

## 1 Method

### 1.1 Hardware Design

**Fig 1:**
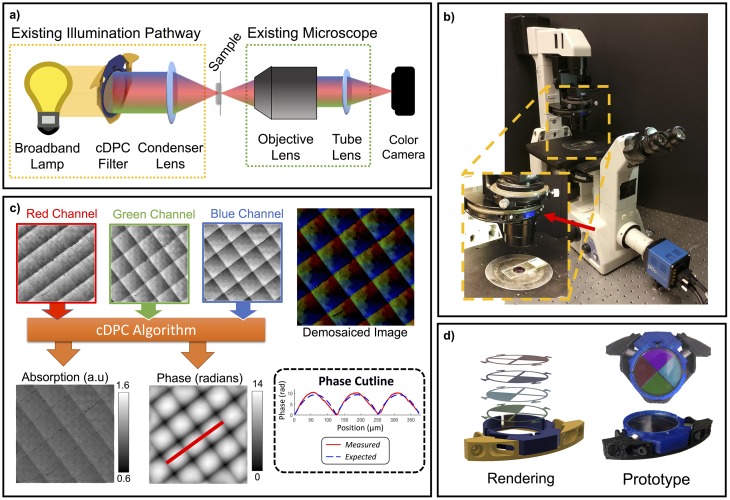
Single-shot color Differential Phase Contrast (cDPC) microscopy. a) Optical schematic of a brightfield microscope with a cDPC color filter placed at the back focal plane of the condenser in Köhler configuration. b) Installation in Nikon TE300 microscope condenser turret. c) Reconstruction: the captured color image is separated into its RGB components, which are then used to recover two unknowns (amplitude and phase) via a well-posed linear deconvolution. The sample is a micro-lens array (Fresnel Technologies 605). d) CAD model and image of fabricated cDPC insert.

As in conventional DPC, our method requires measurements of the sample illuminated by known asymmetric sources. However, in our case the 3 half-circle sources are turned on simultaneously (for example, in each of the three color channels of an LED array). Since we no longer need dynamic source patterning, we need not replace the entire illumination unit of the microscope with an LED array. Instead, we make use of the microscope’s existing condenser unit, which has a turret commonly used for phase contrast inserts or DIC prisms. This intermediate plane can usually be accessed easily by removing the mechanical inserts. Here, we introduce a simple 3D printed color filter that is placed in the condenser turret of a Nikon TE300 microscope (Fig 1A).

Our filter prototype consists of Polyethylene Terephthalate (PET) color filters (Lee Filter, Inc.) which were laser cut to size and installed into a 3D printed insert designed to fit our microscope. Narrow bandwidth illumination filters (e.g. multi-layer coated glass) would provide better spectral selectivity, but suffer from low light throughput and high cost. Therefore, we choose the inexpensive and easy-to-cut PET film filters; the resulting cross-talk between color channels will be accounted for in post-processing, described below.

The total cost of raw materials is approximately $30 and filters were produced quickly with a 3D printer and laser cutter. One filter is shown in Fig 1B; it was installed in the condenser turret of our microscope (Fig 1C), replacing one of the removable phase contrast (Ph1, Ph2 or Ph3) inserts. CAD models of the prototype are included in S3 File.

### 1.2 Calibration

Ideally, our color filters would provide perfect separation of the three source patterns into the three color channels. In reality, both the illumination and camera color channels have cross-talk between the desired wavelengths. To account for this, we separate our calibration into two separate steps: detection-side and illumination-side.

**Fig 2:**
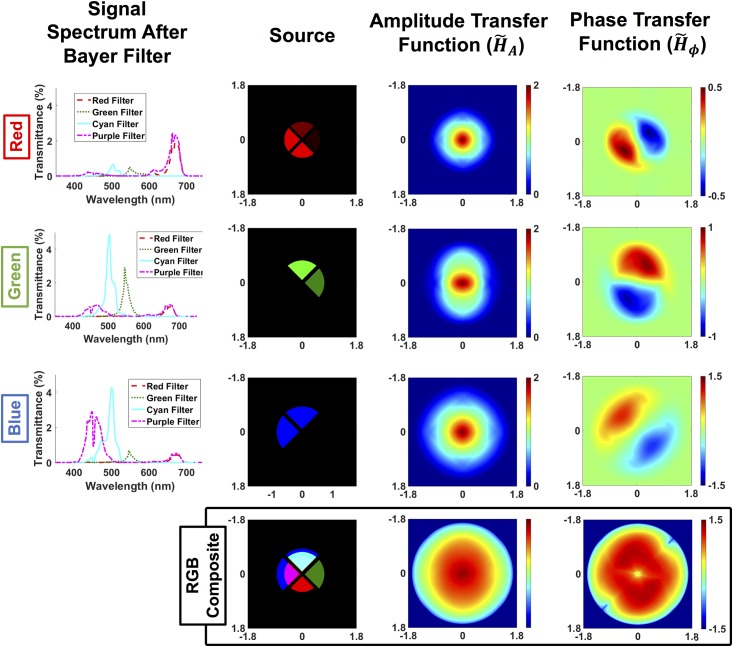
Transfer functions for amplitude and phase contrast in each cDPC color channel. Left: spectral contribution of each illumination filter as captured by the camera’s Bayer pattern. The following columns show the components of the source represented in each image, and the amplitude and phase transfer functions in the spatial frequency domain. Bottom row: sum of each column, representing the calibrated and scaled source and the total coverage of amplitude and phase transfer functions, respectively.

Illumination-side calibration corrects for the relative spectral transmittance of each of the source color filters. The illumination pattern simultaneously encodes three half-circle sources, one each for the RGB color channels. Red and green are opposite half-circles, and blue is rotated by 90 degrees relative to the others. Where the blue and green patterns overlap, a cyan filter (blue + green) was used. Where the blue and red patterns overlap, a purple filter (blue + red) was used. Hence, the final filter design actually contains four quadrants having red, green, cyan and purple filters (see Fig 2).

When filtered by the sensor Bayer pattern, our spectrum bases are not orthogonal. This can be seen in the spectra of each PET film after capture with our color camera (left column of Fig 2). The result is an undesirable loss of asymmetry in the source that reduces phase SNR. We can, however, account for the asymmetry during our reconstruction by modeling the source patterns as in Fig 2.

Detection-side calibration accounts for spectral cross-talk of the camera color channels. Standard RGB Bayer filters do not provide perfect discrimination between RGB wavelengths, but coupling artifacts can be removed by calibration. Given the pixel values from the raw color image with an RGBG Bayer filter (*I*_*r*_, *I*_*g*1_, *I*_*g*2_, *I*_*b*_), we solve for the decoupled color image (*I*_*R*_, *I*_*G*_, *I*_*B*_) that would be obtained if the sample were illuminated with a single color, according to the following equation,

$$\left[\begin{array}{c} I_{r}\\ I_{g1}\\ I_{g2}\\ I_{b} \end{array}\right]=C\;\left[\begin{array}{c} I_{R}\\ I_{G}\\ I_{B} \end{array}\right].$$ (1)

The matrix *C* is a 4×3 calibration matrix describing the coupling between each color channel. It is generated by filtering the broadband source with each filter independently, then measuring the relative red (*I*_*R*_), green (*I*_*G*_) and blue (*I*_*B*_) read-outs to populate the corresponding column vectors of the *C* matrix. The ratio between the intensities of each flat-field image at each detection channel provides a linear weighting of the contribution of each source to our color measurement. Once *C* has been measured once, it can be used to pre-process all later measurements by solving Eq (1). This step is important for reducing artifacts in the phase results.

Another important step for cDPC is to account for wavelength-dependent changes in phase and spatial frequency. DPC recovers absorption (*μ*) and phase (*ϕ*) information from intensity measurements. These quantities are defined as:

$$\mu =\frac{2\pi }{\lambda_{0}}\alpha d,\;\phi =\frac{2\pi }{\lambda_{0}}nd,$$ (2)

where *λ*_0_ is a reference wavelength, *d* is the thickness of the sample, *n* represents refractive index and *α* indicates absorption coefficient. Absorption and phase transfer functions are determined by illumination numerical aperture (NA), objective NA and illumination wavelength [17]. In our proposed color-multiplexed DPC, the transfer functions must also consider the change in wavelength of each color channel. Phase (*ϕ*) depends on which wavelength is used. By assuming no dispersion in the sample, we can use Eq (2) to synthesize phase for any wavelength by simply multiplying the optical path length (*nd*) by the wave number ($\frac{2\pi }{\lambda_{0}}$) of a desired reference wavelength *λ*_0_.

### 1.3 Forward model

We linearize our forward model by deriving the Weak-Object Transfer Functions (WOTFs) for both amplitude and phase [14, 17, 26]. The WOTF formulation linearizes phase recovery by neglecting the nonlinear scatter-scatter term; this is a good approximation when the object is weak (having low absolute phase or amplitude). Each image is modeled as the sum of convolutions between color-dependent point spread functions (PSFs) for intensity, and physical quantities—absorption and phase (*μ*, *ϕ*),

$$I\left(\vec{r},\lambda \right)=I_{0}\left(\lambda \right)+H_{\mu }\left(\vec{r},\lambda \right)⊗\mu \left(\vec{r}\right)+i\cdot H_{\phi }\left(\vec{r},\lambda \right)⊗\phi \left(\vec{r}\right),$$ (3)

where $\vec{r}$ represents 2D real-space coordinates, *I* is the color intensity measurement, *I*_0_ is the background signal, ⊗ denotes convolution, *H*_*μ*_ and *H*_*ϕ*_ are PSFs for absorption and phase, respectively. Taking the 2D Fourier transform of both sides of Eq 3, we obtain:

$$\tilde{I}\left(\vec{f},\lambda \right)={\tilde{I}}_{0}\left(\lambda \right)\cdot \delta \left(\vec{f}\right)+{\tilde{H}}_{\mu }\left(\vec{f},\lambda \right)\cdot \tilde{\mu }\left(\vec{f}\right)+i\cdot {\tilde{H}}_{\phi }\left(\vec{f},\lambda \right)\cdot \tilde{\phi }\left(\vec{f}\right),$$ (4)

where $\vec{f}$ is 2D spatial frequency coordinates, $\tilde{\cdot }$ denotes Fourier transform, ${\tilde{H}}_{\mu }$ and ${\tilde{H}}_{\phi }$ are the wavelength-dependent transfer functions for absorption and phase, respectively. Given a known source (*S*), and pupil function (*P*) which we model as a circle with radius set by the objective NA and wavelength *λ*, the transfer functions are [17, 26]:

$${\tilde{H}}_{\mu }\left(\vec{f},\lambda \right)=\left[P\left(\vec{f},\lambda \right)⋆\left(P\left(\vec{f},\lambda \right)\cdot S\left(-\vec{f},\lambda \right)\right)+\left(P\left(\vec{f},\lambda \right)\cdot S\left(-\vec{f},\lambda \right)\right)⋆P\left(\vec{f},\lambda \right)\right]$$ (5)

$${\tilde{H}}_{\phi }\left(\vec{f},\lambda \right)=\frac{\lambda_{0}}{\lambda }\cdot \left[P\left(\vec{f},\lambda \right)⋆\left(P\left(\vec{f},\lambda \right)\cdot S\left(-\vec{f},\lambda \right)\right)-\left(P\left(\vec{f},\lambda \right)\cdot S\left(-\vec{f},\lambda \right)\right)⋆P\left(\vec{f},\lambda \right)\right],$$ (6)

where ⋆ denotes cross-correlation. Note that because spatial frequency is a function of wavelength, the source shape *S*(*λ*) and pupil function *P*(*λ*) also depend on wavelength. Specifically, the diameters of the source and transfer functions in Fourier space are inversely proportional to the wavelength of the color channel. Hence, blue illumination provides larger Fourier space coverage and better resolution than red. Our forward model accounts for these differences in the color channel’s transfer function. Fig 2 shows the absorption and phase transfer functions for *λ* = 450*nm*, *λ* = 546*nm* and *λ* = 670*nm*, with top-right, bottom-right and top-left half-circle sources, respectively.

Examining Fig 2, we see that the absorption transfer functions for each color channel are symmetric low-pass filters. The phase transfer functions, on the other hand, are asymmetric band-pass-like filters with a line of missing frequencies along the axis of asymmetry. By rotating the blue half-circle by 90 degrees relative to the red and green ones, we fill in the missing line. The overall amplitude and phase transfer functions for cDPC are shown in the last row of Fig 2, calculated by summing the absolute values of each color transfer function. As with previous DPC implementations, absorption information loses contrast at high spatial frequencies. Phase has a similar drop-off at high frequencies, but also loses contrast in the low spatial frequency regions. Hence, SNR will be important for accurately recovering low-frequency phase information. The maximum spatial frequency range captured is 2× the NA of the blue color channel. However, the final resolution using cDPC is set by the diffraction limit of green light, since our total frequency coverage is set by the maximum spatial frequency which is measured by *two or more* color channels. This comes as an implication of trying to recover two unknowns, amplitude and phase, thus requiring at least two measurements.

### 1.4 Inverse problem

Using the forward model developed in Section 1.3, our inverse problem aims to minimize the difference between the measured color image and that which would be measured, given the estimate of the sample’s amplitude and phase:

$$\begin{array}{c} \min_{\mu ,\phi }\sum_{m=1}^{3}\frac{1}{2}∥{\tilde{I}}^{\prime }\left(\lambda_{m}\right)-{\tilde{H}}_{\mu }\left(\lambda_{m}\right)\cdot \tilde{\mu }-i\cdot {\tilde{H}}_{\phi }\left(\lambda_{m}\right)\cdot \tilde{\phi }{∥}_{2}^{2}+R\left(\mu ,\phi \right), \end{array}$$ (7)

where ${\tilde{I}}^{\prime }$ is the spatial frequency spectrum of the background-subtracted intensity, *m* is the wavelength index and *R*(*μ*, *ϕ*) is a regularization term (typically on the order of 10^−3^). This problem is linear and can be solved with a one-step least-square solution (e.g. Wiener deconvolution [27]) or by an iterative algorithm (e.g. gradient descent). The ideal choice of regularizer *R*(*μ*, *ϕ*) depends on the sample and noise. Basic *ℓ*_2_ regularization should be tuned to suppress noise amplification in spatial frequencies that are measured with low-contrast, without destroying sample information at those frequencies. Alternatively, if the sample is sparse (only a few non-zero values), one can use an *ℓ*_1_ regularizer [28]. Other types of *a priori* information may be incorporated by appropriate regularization. In our experiments, we make no assumptions on the sample and so use *ℓ*_2_ regularization. Eq (7) thus becomes,

$$\begin{array}{c} \min_{\mu ,\phi }\sum_{m=1}^{3}\frac{1}{2}∥{\tilde{I}}^{\prime }\left(\lambda_{m}\right)-{\tilde{H}}_{\mu }\left(\lambda_{m}\right)\cdot \tilde{\mu }-i\cdot {\tilde{H}}_{\phi }\left(\lambda_{m}\right)\cdot \tilde{\phi }{∥}_{2}^{2}+\gamma_{\mu }\cdot ∥\mu {∥}_{2}^{2}+\gamma_{\phi }\cdot ∥\phi {∥}_{2}^{2}, \end{array}$$ (8)

which remains differentiable and allows us to find the global minimum solution for absorption and phase with a single matrix inversion step. The final reconstruction for the absorption and phase maps can therefore be written mathematically as:

$$\mu =F^{-1}\;\left\{\frac{\left(\sum_{m}{\left|{\tilde{H}}_{\phi ,m}\right|}^{2}+\gamma_{\phi }\right)\cdot \sum_{m}\left({\tilde{H}}_{\mu ,m}^{*}\cdot {\tilde{I}}_{m}^{\prime }\right)-\sum_{m}\left({\tilde{H}}_{\mu ,m}^{*}\cdot {\tilde{H}}_{\phi ,m}\right)\cdot \sum_{m}\left({\tilde{H}}_{\phi ,m}^{*}\cdot {\tilde{I}}_{m}^{\prime }\right)}{\left(\sum_{m}{\left|{\tilde{H}}_{\mu ,m}\right|}^{2}+\gamma_{\mu }\right)\cdot \left(\sum_{m}{\left|{\tilde{H}}_{\phi ,m}\right|}^{2}+\gamma_{\phi }\right)-\sum_{m}\left({\tilde{H}}_{\mu ,m}\cdot {\tilde{H}}_{\phi ,m}^{*}\right)\cdot \sum_{m}\left({\tilde{H}}_{\mu ,m}^{*}\cdot {\tilde{H}}_{\phi ,m}\right)}\right\}$$ (9)

$$\phi =F^{-1}\;\left\{\frac{-i\cdot \left[\left(\sum_{m}{\left|{\tilde{H}}_{\mu ,m}\right|}^{2}+\gamma_{\mu }\right)\cdot \sum_{m}\left({\tilde{H}}_{\phi ,m}^{*}\cdot {\tilde{I}}_{m}^{\prime }\right)-\sum_{m}\left({\tilde{H}}_{\mu ,m}\cdot {\tilde{H}}_{\phi ,m}^{*}\right)\cdot \sum_{m}\left({\tilde{H}}_{\mu ,m}^{*}\cdot {\tilde{I}}_{m}^{\prime }\right)\right]}{\left(\sum_{m}{\left|{\tilde{H}}_{\mu ,m}\right|}^{2}+\gamma_{\mu }\right)\cdot \left(\sum_{m}{\left|{\tilde{H}}_{\phi ,m}\right|}^{2}+\gamma_{\phi }\right)-\sum_{m}\left({\tilde{H}}_{\mu ,m}\cdot {\tilde{H}}_{\phi ,m}^{*}\right)\cdot \sum_{m}\left({\tilde{H}}_{\mu ,m}^{*}\cdot {\tilde{H}}_{\phi ,m}\right)}\right\},$$ (10)

where ⋅ represents point-wise matrix multiplication, *γ*_*μ*_ and *γ*_*ϕ*_ are regularization coefficients of absorption and phase, respectively, and *F*^−1^ denotes the inverse DFT operation. To compute amplitude (*A*) from absorption, we use the relation *A* = *e*^*μ*^, which is similar to the reconstruction method used in [17] but does not assume a pure phase object, leading to additional terms in Eq 10.

## 2 Results and Discussion

**Fig 3:**
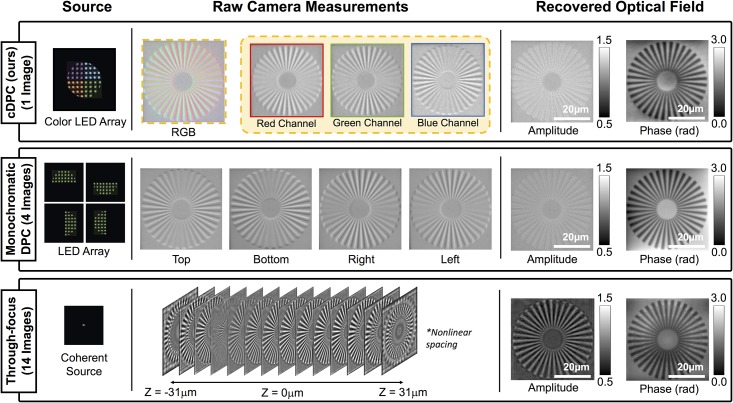
Experimental comparison of single-shot cDPC with monochromatic DPC and through-focus phase retrieval methods. (Left) Source patterns. (Middle) Raw camera measurements. (Right) Recovered optical field. DPC methods (partially coherent) were acquired using a 20× 0.4 NA objective lens, while through-focus images (spatially coherent) were captured using 60× 0.8 NA, in order to ensure equal resolution in all cases.

To experimentally validate our cDPC method, we compare our results with two established QPI methods: monochromatic DPC and through-focus phase retrieval (Fig 3). For fair comparison, all are implemented on the same Nikon TE300 microscope using illumination generated by an RGB LED array (Adafruit). cDPC uses a discretized version of our color filter design displayed on the LED array. Monochromatic DPC uses 4 images captured with each of 4 asymmetric source patterns [18]. Through-focus phase imaging uses only the central green LED (for temporal and spatial coherence) while capturing 14 images at different focus depths; phase is then recovered by a nonlinear optimization phase retrieval method [29].

Because of the coherent illumination, through-focus phase imaging has 2× worse resolution than DPC methods. Thus, we use a 20× 0.4 NA objective lens for DPC methods but switch to a 60× 0.8 NA objective for through-focus phase, in order keep resolution equal for all three. Spatial resolution is quantified using a spoke-pattern phase target [30].

As can be seen in Fig 3, the RGB color channel images have similar contrast to the left, right and top images of the monochromatic DPC, as expected. The phase results are also similar, with equivalent spatial resolution. Because the cDPC image is captured in one shot with color filters, it has lower SNR than monochromatic DPC and deviates in its low-frequency fluctuations, which have weaker transfer function values. Overall, however, single-shot cDPC performs comparably to multi-shot DPC.

**Fig 4:**
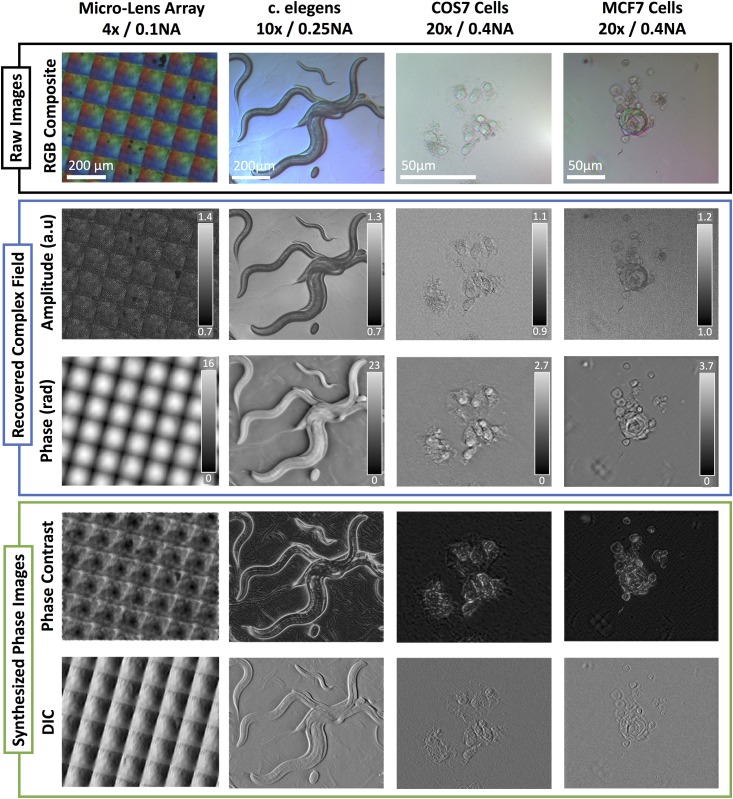
Raw data, phase and amplitude reconstructions, synthesized phase contrast and DIC images for various samples and magnifications: micro-lens array (4x 0.1 NA), wild-type c. elegans (10x 0.25 NA), HEK 293T cells (20× 0.4 NA), MCF7 cells (20× 0.4 NA).

Next, we removed the LED array and reinstalled the microscope’s condenser unit with an broadband arc lamp light source. Alternatively, a high-power blue-phosphor static LED source could be used. We then installed the color filter insert shown in Fig 1C into the condenser turret. Fig 4 shows amplitude and phase reconstructions from our cDPC method with objectives of various magnification, as well as simulated phase contrast and DIC images. Our method is compatible with any standard objective having *NA*_*objective*_ ≤ *NA*_*condenser*_. The ratio of *NA*_*objective*_ to *NA*_*condenser*_ is referred to as the spatial coherence factor *σ* [17], defined as:

$$\sigma =\frac{NA_{condenser}}{NA_{objective}}.$$ (11)

In other words, *σ* < 1 will result in reduced phase contrast as compared to the *σ* ≥ 1 case, as shown in [17]. This is because low frequencies in phase are revealed only when using high-angle illumination in our model. It is important to note that illumination with *σ* > 1 does not improve resolution beyond the incoherent resolution limit, but also does not degrade image quality, allowing us to fix the condenser NA and use any objective with *NA*_*objective*_ ≤ *NA*_*condenser*_ without changing the hardware.

The Nikon TE300 microscope used in this study was configured with a 0.53 NA condenser lens. Imaging with a higher objective NA would require high-NA illumination (e.g. by using a domed LED array [22]). Temporal coherence is set by the bandwidth of the color filters, since these have narrower bandwidth than the camera filters. The full-width-half-maximum (FWHM) bandwidth for our filters was approximately 50nm, which is similar to the emission spectrum of the LED array used previously [17].

### 2.1 Temporal Resolution

**Fig 5:**
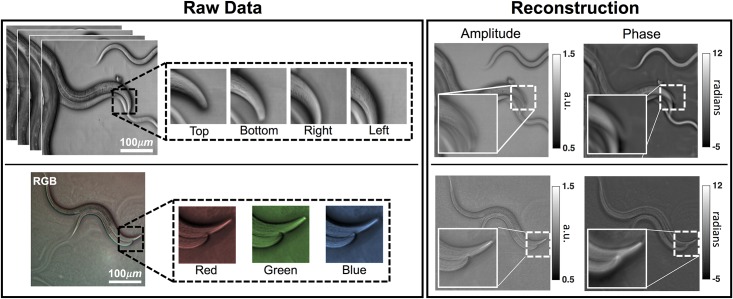
Experimental demonstration of motion blur reduction with cDPC vs. conventional DPC. Our cDPC method results in significantly reduced motion blur artifacts due to its single-shot acquisition.

Since cDPC is single-shot, temporal resolution is set by the camera’s frame rate, giving a factor of 4 improvement over conventional DPC. Single-shot methods reduce artifacts due to motion blur and image registration. This can be seen in Fig 5, where we compare cDPC and conventional DPC (4 images) results for a live c. elegans culture. Motion blur is significantly reduced with cDPC, since the sample changes rapidly between frames, even at 12.5 frames per second. Live sample videos are presented in S1 and S2 Files showing results for both moving c. elegans and cells in a microfluidic channel at 100 FPS, 8× faster than in [18].

### 2.2 Synthesized PhC and DIC Images

**Fig 6:**
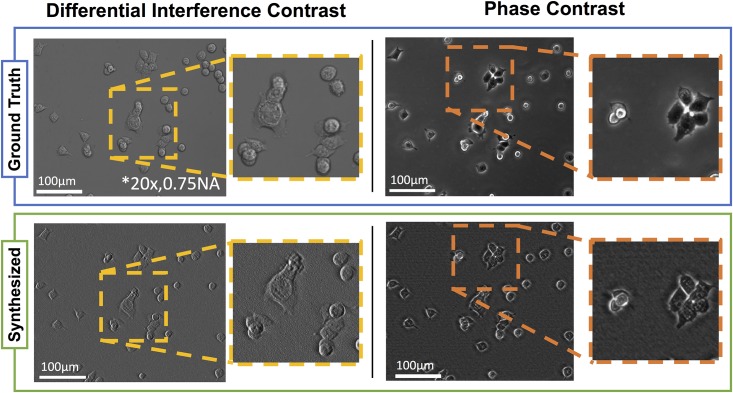
Comparison of standard DIC and PhC images to their synthesized counterparts from cDPC. Ground truth DIC images were acquired using a 20x 0.75 NA objective and phase contrast images using a 20x 0.4 NA PhC objective. cDPC images were acquired using a 20x 0.4 NA objective and our filter insert.

Differential Interference Contrast (DIC) and conventional Phase Contrast (PhC) microscopy are widely used in medicine and biomedical research. Optical components required for their implementation remain expensive, however, and alignment by an experienced user is required for acceptable performance. Both DIC and phase contrast can be described by forward models which produce a qualitative mixture of amplitude and phase images [31, 32]. Quantitative phase imaging methods can therefore be used to synthesize these contrast mechanisms digitally, mimicking the physical optical system through numerical simulation. Synthesized images from cDPC, as well as ground truth DIC and PhC images, are shown in Fig 6 to be comparable.

Synthesizing DIC and PhC may be useful for clinicians and researchers who have been trained with them. While all QPI methods can be used to synthesize these images, our method is particularly well-suited since it is single-shot, allowing for real-time digital synthesis. In addition to further providing quantitative phase, our method is much cheaper to implement than either DIC or PhC, since it requires only the addition of an inexpensive color filter insert and no specialized objectives.

### 2.3 Stained and Dispersive Samples

Our method uses color multiplexing to recover complex-field, making an inherent assumption that the sample is both non-dispersive and colorless. Non-dispersive means that the refractive index does not change appreciably with wavelength:

$$\phi \left(n\left(\lambda \right),d,\lambda \right)\approx \phi \left(n_{0},d,\lambda \right).$$ (12)

This assumption implies that the optical path length (*OPL* = *nd*) will remain constant for all measurements. The relative phase delay will always vary with *λ* (Eq 2), but this is accounted for in our algorithm by scaling our transfer functions based on the relative wavelength of each color channel. Unless the dispersion curve is known and the material is assumed to be uniform, we cannot account for dispersive effects in the sample using the proposed algorithm.

The second assumption is that the sample is colorless, meaning that the absorption does not have chromatic dependence:

$$\mu \left(\lambda \right)\approx \mu_{0}.$$ (13)

This is generally valid for unstained biological samples, which are transparent. Color variations due to filter transmission coefficients at different wavelengths are present, but can be removed by the calibration procedure described in Section 1.2. Color-dependent absorption, such as that created by stained samples, cannot be recovered and will cause errors in the phase result. In practice, these assumptions limit the applicability of our method to unstained uncolored samples. However, quantitative phase reveals the mechanical structure of the microenvironment with high contrast, which may eliminate the need for staining in many applications.

## 3 Conclusion

We have presented a single-shot method for quantitative phase and amplitude imaging based on partially-coherent multiplexed color illumination. The inverse algorithm uses a linear approximation to enable fast reconstruction by deconvolution. Our hardware requirements are simple, inexpensive and compatible with most commercial microscopes; we require only a color camera and a color filter insert placed at the back focal plane of the condenser lens. Unlike phase contrast and DIC, our method does not require special objectives or prisms, reducing hardware costs significantly. We can use the recovered complex-field to synthesize phase contrast and DIC images digitally, matching the functionality of existing systems at a fraction of the cost. Because we assume that samples are non-dispersive and unstained, our method should be used as an alternative, not in conjunction with, chemical staining.

## Supporting Information

S1 File Video of c.elegans. Raw RGB data, recovered quantitative phase and amplitude of live *in vitro* wild-type c.elegans sample. Objective is 10×, 0.25 NA.
(ZIP)

S2 File Video of MCF10a cells in a micro-fluidic channel. Raw RGB data, recovered quantitative phase and amplitude of live *in vitro* MCF10a cells flowing in a microfluidic channel. Objective is 20×, 0.4 NA.
(ZIP)

S3 File 3D models, color filter templates, and processing code for designing and building a cDPC filter. (ZIP)
