# Supplementary figures and images for: Single-shot quantitative phase microscopy with color-multiplexed differential phase contrast (cDPC)
Source: PLoS One. 2017 Feb 2;12(2):e0171228. doi: 10.1371/journal.pone.0171228 (PMC5289592; doi:10.1371/journal.pone.0171228)

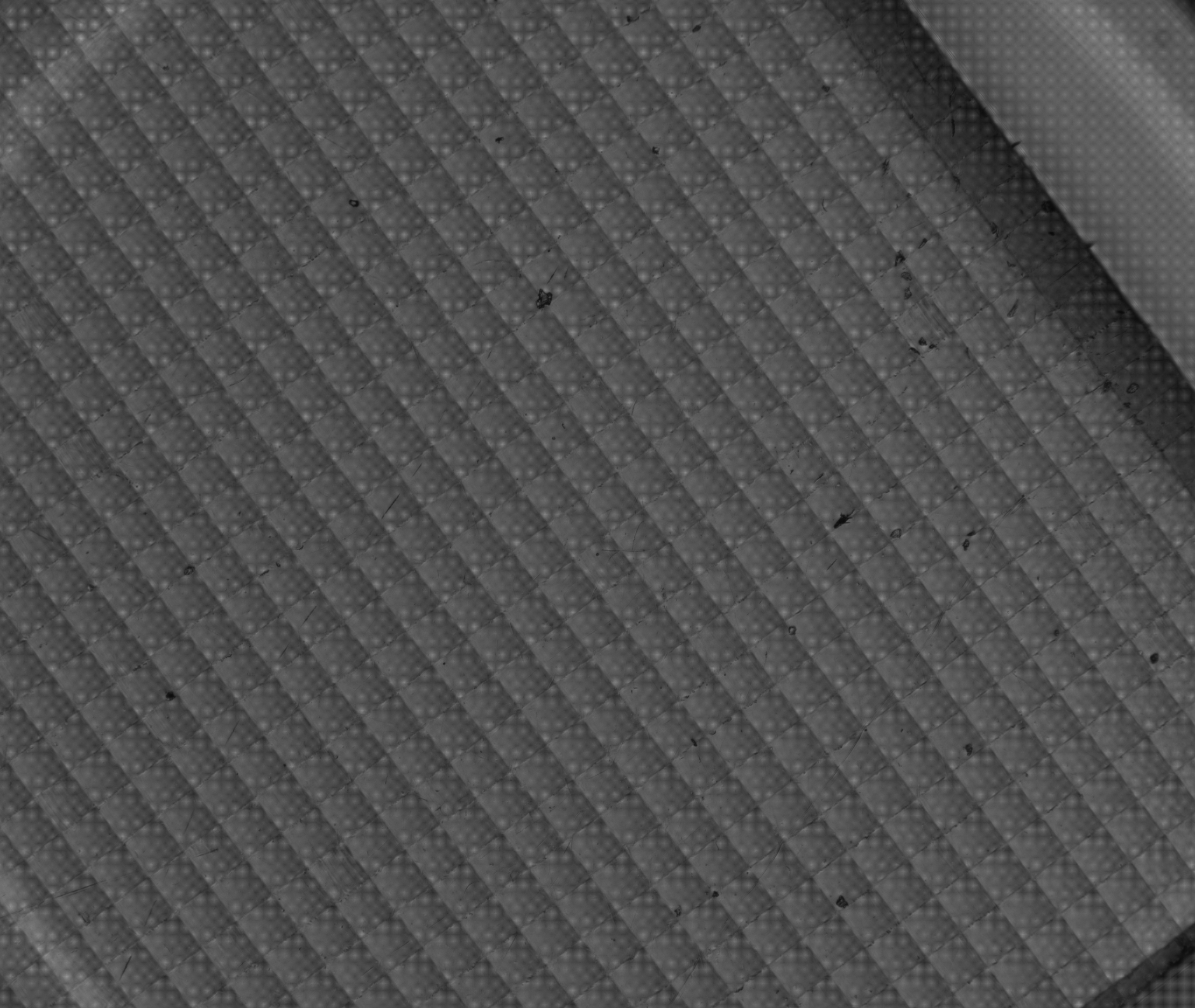

Supplement: S3 File — (ZIP) [file pone.0171228.s003.zip › Public/Code/rgb.tif]
